# 

**Published:** 1853-04

**Authors:** 


### THE COMMENCEMENT EXERCISES OF THE OHIO COLLEGE OF DENTAL SURGERY.

This institution closed its eighth annual course of Lectures on the 19th of February last. The session was one of more than ordinary interest, and the Class larger than at any former session. The committee of examination appointed by the College association, expressed themselves as very much gratified with the satisfactory manner, which the young Gentlemen passed the ordeal of examination. The degrees (owing to the indisposition of the Rev'd. Dr. Aydalotte, President of the board of Trustees,) was conferred by Rev'd. Dr. S. W. Fisher, who also addressed the graduates in a very felicitous manner. The address was peculiarly appropriate. The valedictory address was by the Dean of the Faculty. Subject—The Evidences of Wisdom as Displayed in the Development of the Teeth. The degree of Doctor of Dental Surgery was conferred on the following gentlemen.

| | |
|---|---|
| Joseph Richardson, | Cincinnati, O. |
| James T. Irwin, | " " |
| J. M. Lewis, | Marion, Ills. |
| H. A. McDaniel, | Kentucky. |
| H. E. Eames, | Rome, N. Y. |
| J. F. Johnson, | Indianapolis, Ia. |
| John Harris, | Salem, Ohio. |
| S. D. Muse, | Sharon, Mississippi. |

The meetings of the College Association and Mississippi Valley Association, passed off very pleasantly, and there was a larger number of Dentists in the city during the week than ever before.

### OHIO COLLEGE OF DENTAL SURGERY.

By reference to the proceedings of the College Association it will be seen that Prof. G. Mendenhall has resigned his Chair. This was rendered necessary by his acceptance of the Chair of Obstetrics in the Miami Medical College It is with much regret that we part with the Dr., who has by his kind and gentlemanly manner won the confidence of all the students. The Dr.'s lectures on Pathology and Therapeutics have always been interesting and instructive, his main object has been to impart useful knowledge, and in this he has succeeded. Dr. Smith who has been lecturing in his place during the past winter with entire satisfaction to the Class, is now appointed to the same chair, and will enter on its duties with zeal and ability.

Dr. Van Emon who has been demonstrator of mechanical Dentistry, for the last two years finding his own office duties increasing on his hands; was unable to devote that amount of time which appeared to be necessary to this department—resigned also. And the College Association thought best to recommond this department as a professorship. This change has been made, leaving the chair of Prof. J. Allen, Operative Dentistry, which he has heretofore filled. Dr. H. R. Smith of Terra Haute, Ia., a gentleman of experience and of reputation, was selected and has been appointed to the Chair of Mechanical Dentistry He designs spending his entire time with the class, except indeed so far as their time may be occupied in hearing the lectures of the other Professors.

The organization is one which cannot fail to commend itself to the profession.

### PHILADELPHIA COLLEGE OF DENTAL SURGERY AND NEW YORK COLLEGE OF DENTAL SURGERY.

We have received notices of the commencement in both of these Institutions, and are pleased to learn that both have made a fair and advantageous beginning. The number of students in attendance at each of our Dental Colleges, has been larger than could have been expected, and certainly speaks well for the estimation in which they are begining to be held by the Profession. We hope and believe that they will exert an influence for great good and soon rank with our best medical schools of the land.

The Baltimore School has, we presume closed by this time a very prosperous session. It would have afforded us much pleasure to have been present at their commencement.

Subscribers are informed that remittances if made in good current funds are at our risk—and letters should be addressed to "Dental Register," or Dr. James Taylor 4th street, Cincinnati, Ohio.

### NINTH ANNUAL ANNOUNCEMENT
OF
### THE OHIO COLLEGE OF DENTAL SURGERY.
**Session of 1853–'54.**

The regular course of lectures in this institution, commences the first Monday of November, and closes on the third Wednesday of February.

**FACULTY.**

JAMES TAYLOR, M. D., D. D. S.
*Professor of the Principles and Practice of Dental Surgery.*

THOMAS WOOD, M. D.,
*Professor of Anatomy and Physiology.*

J. B. SMITH, M. D.,
*Professor of Pathology and Therapeutics.*

JOHN ALLEN, D. D. S.,
*Professor of Operative Dentistry.*

H. R. SMITH, D. D. S.
*Professor of Mechanical Dentistry.*

**EXAMINING COMMITTEE.**

DENTAL.

A. S. Talbert, D. D. S., of Lexington, Ky.
W. H. Goddard, D. D. S., of Louisville, Ky.
H. E. Peebles, D. D. S., of Lexington, Mo.

MEDICAL.

Prof. Mendenhall, M. D., of Cincinnati, O.
Israel Dodge, M. D., of Cincinnati, O.

The Faculty in issuing this their Ninth Annual Announcement, take pleasure in stating to the Profession that the Institution is based upon a firm foundation, being chartered by the state and owned and governed by stockholders (most of whom are regular Dental Practitioners) in connection with a board of Trustees. In the reorganization of the Faculty, the Profession have every assurance that the interest of the school has been consulted and that the course of instruction will be such as to meet the wants of the dental student.

A building suitable in all the conveniences for a Dental College is owned by the Profession and dedicated to the advancement of dental science.

The library is increasing in useful and appropriate books, and a fund is secured for the continued increase of this and such apparatus as may be needed.

The Professor of Mechanical Dentistry has made arrangements to open the laboratory and infirmary, by the middle of October. Every facility will be afforded the student, and every thing requisite furnished, but such materials as are of a perishable nature.

Tickets for the course, $100,00 ; Matriculating Ticket, 5,00 ; Diploma Fee, $25,00. Good board can be had at from $2,50 to $3,00 per week.

JAMES TAYLOR, *Dean.*

### RECEIPTS FOR THE REGISTER.

L. W. Frery, Bellvue, O., vol. 6,..... $1
B. A. Kenedy, Wilmington N. C. vol. 6, 2
C. R. Sterneman, Gallipolis, O., vol. 6, 1
A. P. Mackey, Lexington, Mo., vol. 6,.. 2
T. Hinson, Lexington, Mo., vol. 6,...... 2
A. G. Major, Booneville, Mo., vol. 6,... 2
C. H. Cook, Madison, Wis., vol. 6,..... 2
E. H. Quinlan, Chicago, Ill., vol. 6,.... 2
H. A. Beamer, Sharpsburg, Ky., vol. 6, 2
C. O. Cone, Baltimore, Md., vol's. 4, 5, and $1 on 6,.......... 5
E. B. Welsh, Wilmington, O., vol's. 4, and 5,.......... 4
M. DeCamp, Mansfield, O, vol. 6,...... 2
C. P. Culver, New Castle, Ky., vol. 6,.. 2
James B. Wiiliams, Monogahela, Pa., vol. 6,.......... 2
H. W. Robbins, Ludlow, Miss., vol. 5,. 2
J. P. Norman, Keokuk, Iowa, vol. 5,... 2
T. R. Davis, Rasco, Ky., vol. 5,....... 2
H. Barron, St. Louis, Mo., vol. 6,...... 2
J. W. Keyes, Quincy, Florida, vol. 5&6, 4
E. Bray, Evensville, Ia., v ol.6,........ 2
Nimrod Hutt, Willsbore, O., vol. 6,.... 2
J. McClure, Carrolton, Miss., " 5 6 & 7, 5
M. N. Manlove, Logansport, Ia., "1 2 & 5, 5
J. A. Doughirty, Bowlin Green, Ky., vol. 4,.......... 2
G. Watt, Xenia, O, vol. 6,.......... 2
S. F. Compton, Hillsboro, O, vol. 6,.... 2
B. Wood, Nashville, Tenn., vol. 6,..... 2
P. Knowlton, Cin., O., vol's. 3 4 & 5,... 6
G. L. Van Eman, Cin., O., vol's 4 & 5,. 4
Eli Collins, Connersville, Ia., vol's. 4 5 and 6,.......... 6
C. Bonsall, Cin., O., vol. 6,.......... 2
W. H. Goddard, Louisville Ky., vol. 6,. 2
E. Hale, St. Louis, Ala., vol's. 3 & 4,... 4
J. Payne, Gallipolis, O., " 3 & 4,... 4
I. C. Howells, Hamilton, O., vol. 6,..... 2
Dr. Edgerly, St Louis, Ala., vol. 4,..... 2
H. E. Peebles, Lexington, Mo., vols. 4 5 and 6,.......... 6
W. M. Hunter, Cin., O., vol's 5 & 6,... 4
H. R. Smith, Terre Haute, Ia., vol. 6,... 2
B. Salterthwaite, Lima, O., vol. 6,..... 2
J. C. Ross, Nashville, Tenn., vol. 3 4 5 and 6,.......... 8
A. N Newton, Richmond, Ia., vols. 3 4 and $1 on 6,.......... 5
Saml. Ayres, Danville, Ky., vol's. 4 5 and 6,.......... 9

